# Shared Psychosis in Family Dynamics: Two Cases of Schizophrenia in Close Relatives

**DOI:** 10.1192/j.eurpsy.2025.2213

**Published:** 2025-08-26

**Authors:** L. Demiraj, E. Sotiri

**Affiliations:** 1 “Mother Teresa” UHC, Tirana, Albania

## Abstract

**Introduction:**

Shared psychotic disorder (folie à deux) is a rare mental health condition where delusions are transmitted from one person to another within a close relationship. Understanding these complex dynamics is crucial for effective diagnosis and treatment.

**Objectives:**

To present two cases of shared psychosis involving close relatives diagnosed with schizophrenia, aiming to open discussion on the complex dynamics of familial relationships and their impact on mental illness.

**Methods:**

2 case reports.

**Results:**

We present two cases of shared psychosis involving close family members. The first case involves two sisters, aged 43 and 48, both diagnosed with paranoid schizophrenia. The 43-year-old sister was admitted following a suicide attempt and exhibited active psychosis with paranoid delusions. During her hospitalization, the older sister displayed identical delusions and refused her sibling’s treatment, leading to her own admission. Both sisters, previously untreated and highly educated, were managed with antipsychotic medications. While the delusions were encapsulated during treatment, concerns remain about their shared living environment and its potential influence on their ongoing recovery.

The second case involves a 33-year-old woman with a decade-long history of schizophrenia who was brought to the emergency room malnourished and uncommunicative, having not eaten for over a week. She was fed via a nasogastric tube during her lengthy hospitalization and placed on a regimen of Clozapine. The patient exhibited severe negative symptoms and behaviors suggestive of auditory hallucinations. Her mother, also of low educational and socioeconomic status, was present throughout her stay but displayed delusional beliefs that interfered with her daughter’s treatment. Following restrictions on the mother’s access to the patient, the daughter showed slight improvement and began eating independently.

**Conclusions:**

These cases highlight the significant impact that familial relationships and shared psychosis can have on the progression and management of schizophrenia. The challenge in treating shared delusions lies not only in addressing the symptoms but also in mitigating the interpersonal dynamics that may perpetuate them. Further investigation into family-based psychosis and its treatment is warranted.

**Disclosure of Interest:**

None Declared

